# We need to talk about adverse events during physical rehabilitation in critical care trials

**DOI:** 10.1016/j.eclinm.2024.102439

**Published:** 2024-02-01

**Authors:** Sabrina Eggmann, Peter Nydahl, Rik Gosselink, Bernie Bissett

**Affiliations:** a Department of Physiotherapy, Inselspital, Bern University Hospital, Bern, Switzerland; b Nursing Research, University Hospital of Schleswig-Holstein, Kiel, Germany; c Department of Rehabilitation Sciences, Faculty of Movement and Rehabilitation Sciences, University Hospitals Leuven, Leuven, Belgium; d Faculty of Health, University of Canberra, Australia; e Canberra Health Services, Canberra, Australia

Physical impairments are a common complication in critical illness survivors, reducing functional independence, quality of life, and return to work. Early physical rehabilitation improves physical function 6 months after discharge,^1^ but a 66% increased chance for adverse events^1^ led to concerns about its safety. In this Commentary, we discuss difficulties with our current reporting of adverse events during physical rehabilitation in critical care trials.

The World Health Organization defines patient safety as “the absence of preventable harm to a patient and reduction of risk of unnecessary harm associated with health care to an acceptable minimum”.^2^ When we apply this to physical rehabilitation in critical care, a conflict appears: immobility causes harm, but so can activity. The most commonly reported adverse events in these trials are transient haemodynamic changes and oxygen desaturation, yet they also frequently occur during routine care.^3^ Haemodynamic and respiratory changes were the main safety concern in two recent trials that reported an increase in adverse events following intensive physical rehabilitation.^4^^,^^5^ Although some of these events required medical intervention, most resolved without further complications with the exception of one cerebrovascular event.^4^ Thus, clearly not all adverse events are equally problematic. Moreover, their aggregation under the banner of adverse events might falsely lead to the perception of a risky intervention deterring clinicians from using a potentially beneficial intervention. Adverse events can be categorised into safety events without harm, events with minor harm, and serious to fatal events (Fig. 1).^6^ For instance, a recent stepped-wedged randomised controlled trial solely reported adverse events with harm (i.e., falls and unplanned extubations).^7^ This delineation in reporting may help to ensure the balance of risk and benefit is appraised fairly when amalgamating evidence.

**Fig. 1:**
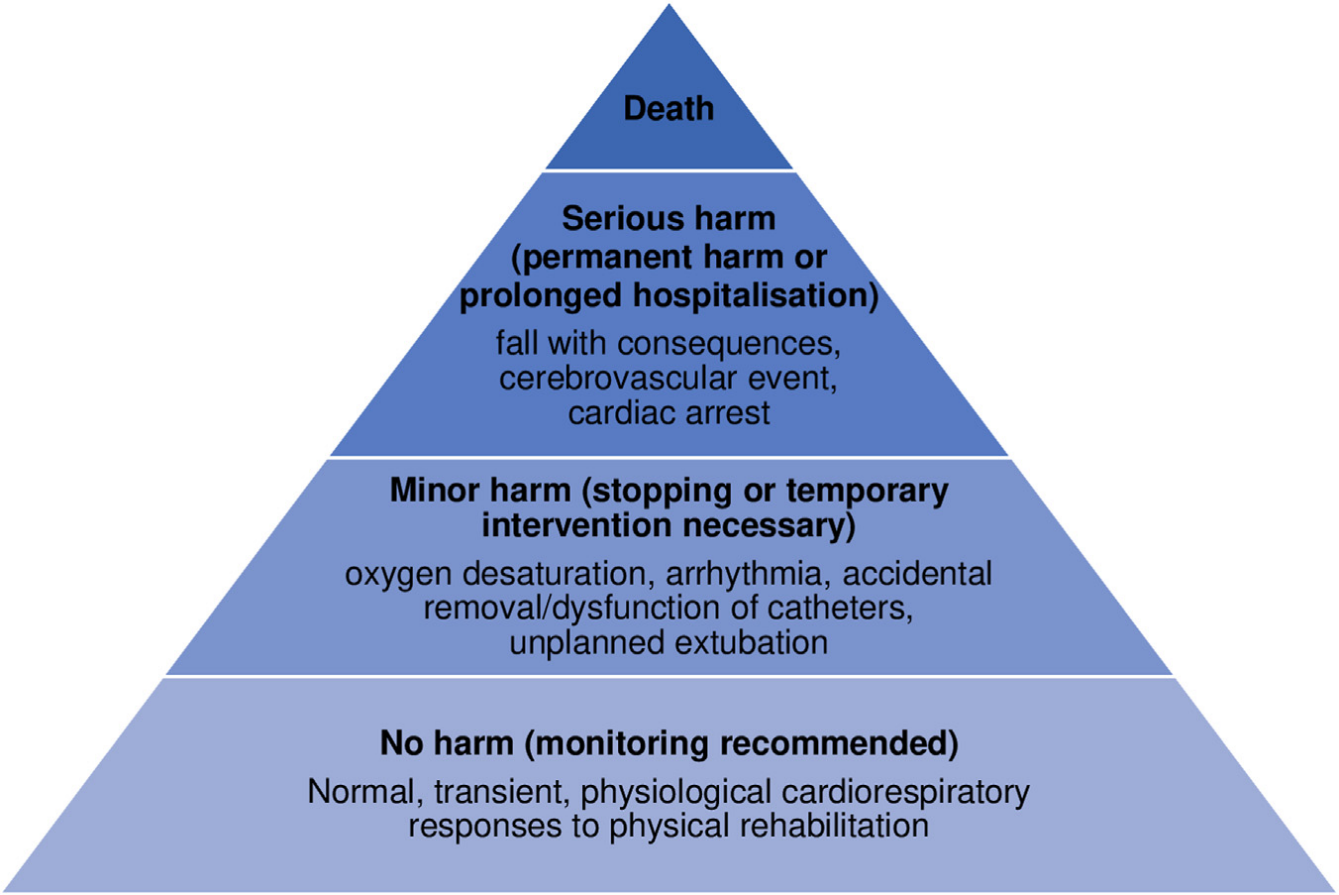
**Frequent safety events during physical rehabilitation in critical care and their categorization**. The occurrence of harm might lead to additional interventions, for example for minor harm: re-insertion of tubes/lines, increasing oxygen, endotracheal suctioning; or for serious harm: cardio-pulmonary resuscitation, additional examinations, invasive procedures, thrombolysis, surgery, or others.

Transient changes in physiological parameters are to be expected during exercise. Physical activity leads to energy expenditure increasing oxygen consumption dependent on the level of intensity. Clearly, an oxygen supply-demand imbalance in patients who are critically ill must be avoided. Nevertheless, classifying transient physiological changes as adverse events in rehabilitation trials could lead to an underdosed intervention whereby physiological reactions are avoided due to overcaution. In consequence, trial results might be inconclusive because of a lack of group separation. Over-reporting of a potentially non-harmful event might therefore inadvertently hinder us from proving effectiveness and contributes to underuse in clinical practice.

We further propose that cardiorespiratory monitoring should primarily be used to target exercise intensity individually during critical care rehabilitation. This is particularly important because we still lack consensus on the appropriate dose—specifically the frequency, intensity and duration—of early rehabilitation interventions. Moreover, cardiorespiratory response analysis found that physiological reactions are highly variable within and across patients who are critically ill.^8^ While extra caution is required in patients who are elderly, obese, or with multiple organ failure, an increased response to exercise might be achieved with active patient participation, shorter and more intense physical rehabilitation interventions.^8^ Judicious monitoring of cardiorespiratory parameters might actually allow a more personalised, adjusted dose of rehabilitation. In this context, cardiorespiratory parameter variation is an inherent aspect of optimal rehabilitation. Hence, deviations from baseline should not generally be labelled as an adverse event unless they persist beyond the activity or necessitate medical intervention (Fig. 1).

Furthermore, there is more to safe monitoring than what we see on the surveillance screen. One of the main concerns of patients engaging with early rehabilitation is exhaustion,^9^ yet this is rarely reported in critical care rehabilitation trials. Clinicians should carefully monitor recovery by ensuring that parameters return to baseline between sets before escalating further interventions. However, exhaustion might also imply a lack of energy. Indeed, muscle wasting in critical illness has been linked with reduced ATP levels suggesting an impaired bioenergetic status in the early phase.^10^ The inability of the muscle to respond to physical rehabilitation interventions therefore need further consideration. Lacking any biomarkers, clinicians should focus on the *quality* of the movement as a vital parameter for fatigability; for example, a patient practising sitting balance with a physiotherapist in ICU can exhibit fatigue by slowly losing head control while sitting on the edge of the hospital bed. Clearly this fatigue is not an adverse event per se, but informs clinician's decision to continue a therapy session or allow rest.

In summary, it is high time to talk about how we define adverse events in critical care rehabilitation studies. Are we focusing on the right parameters? What is the acceptable minimum of harm? Are we overly cautious and fixated on seeing potential harm instead of using cardiorespiratory parameters for a more personalised and targeted approach to optimise dose of early rehabilitation? For now, physical rehabilitation remains a balancing act between contraindications and benefits, over- and underdosing, fatigability and recovery. But we must be careful in not underselling an evidence-based intervention.

## Contributors

All authors conceived the study. SE wrote the first draft, while PN, RG, and BB provided critical input and revised the manuscript. All authors approved the final manuscript.

## Declaration of interests

SE, PN, BB declare no conflict of interest. RG reports Elsevier royalties.
